# The ultrafast, high‐pitch turbo FLASH mode of third‐generation dual‐source CT: Effect of different pitch and corresponding SFOV on image quality in a phantom study

**DOI:** 10.1002/acm2.13466

**Published:** 2021-11-09

**Authors:** Yang Zhou, Lei Hu, Silin Du, Rui Jin, Wangjia Li, Fajin Lv, Zhiwei Zhang

**Affiliations:** ^1^ Department of Radiology the First Affiliated Hospital of Chongqing Medical University Chongqing China; ^2^ Network Information Center the First Affiliated Hospital of Chongqing Medical University Chongqing China

**Keywords:** dual‐source CT, high‐pitch CT, image features, image quality

## Abstract

**Purpose:**

To investigate the effect of different pitches and corresponding scan fields of view (SFOVs) on the image quality in the ultrafast, high‐pitch turbo FLASH mode of the third‐generation dual‐source CT using an anthropomorphic phantom.

**Methods:**

The phantom was scanned using the ultrafast, high‐pitch turbo FLASH protocols of the third‐generation dual‐source CT with the different pitches and corresponding SFOVs (pitches: 1.55 to 3.2 with increments of 0.1, SFOVs: 50 cm to 35.4 cm). The objective parameters such as the CT number, image noises, signal‐to‐noise ratio (SNR), contrast‐to‐noise ratio (CNR), and artifacts index (AI), and image features from the head, chest, and abdomen were compared between the CT images with a pitch of 1.55 and SFOV of Ø 50 cm and a pitch of 3.2 and SFOV of Ø 35.4 cm. Then, the 18 series of CT images of the head, chest, and abdomen were evaluated by three radiologists independently.

**Results:**

The differences in the CT numbers were not statically significant between the CT images with a pitch of 1.55 and SFOV of Ø 50 cm and a pitch of 3.2 and SFOV of Ø 35.4 cm from most body parts and potential combinations (*p *> 0.05), Most of the image noises and the AI from the images with the pitch of 1.55 were significantly lower than those with the pitch of 3.2 (*p *< 0.05), and the SNR and CNR from the images with the pitch of 1.55 were higher than those with the pitch of 3.2. There were significant differences in the first‐order features and texture features of the head (59.3%, 28.3%), chest (66%, 35.7%), and abdomen (71.6%, 64.7%) (*p *< 0.05). The subjective image quality was excellent when the pitch was less than 2.0 and gradually decreased with the increasing pitch. In addition, the image quality decreased significantly when the pitch was higher than 3.0 (all *k*≥0.69), especially in the head and chest.

**Conclusions:**

In the ultrafast, high‐pitch turbo FLASH mode of the third‐generation DSCT, increasing the pitch and lowering the corresponding SFOV will change the image features and cause more artifacts degrading the image quality. Specific to the clinical needs, decreasing the pitch not only can expand the SFOV but also can improve the image quality.

## 1 INTRODUCTION

Second‐generation dual‐source CT(DSCT) FLASH mode scan uses two X‐ray sources and two corresponding data acquisition systems simultaneously and has one quarter of the gantry rotation time to scan with the pitch of 3.2, and the high temporal resolution up to 75 ms. The third‐generation DSCT turbo FLASH mode is equipped with wider detector rows and has a gantry rotation speed of 0.25 s, 737 mm/s feed table, and a temporal resolution up to 66 ms with a pitch of 3.2. The acquisition time of the high‐pitch wide‐coverage scan is significantly reduced, which helps to eliminate the motion artifacts in the cardiovascular application when dealing with the uncooperative or pediatric patients,^1^, ^2^, ^3^, ^4^, ^5^, ^6^, ^7^, ^8^, ^9^, ^10^, ^11^ and to reduce the radiation dose and contrast volume when the high‐pitch low‐voltage protocols are used.^12^, ^13^, ^14^, ^15^, ^16^, ^17^, ^18^, ^19^

The scan field of view (SFOV) of the second‐generation DSCT FLASH mode is limited to 33 cm in diameter (Ø 33 cm), which is not suitable to use in obese patients. In contrast, the SFOV of the third‐generation DSCT turbo FLASH mode can provide a maximum SFOV of 50 cm in diameter with a pitch of 1.55, which greatly expands the scope of clinical use. However, low pitch helical scans provide better image quality in conventional CT.^20^, ^21^ Flohr et al.^22^ found no significant differences in the quantitative measures of the image quality between the single‐source scan with a pitch of 1.0 and the dual‐source scan with a pitch of 3.2 in second‐generation DSCT. But, artifacts were more prevalent in the 3.2 high‐pitch FLASH mode head scan. Nevertheless, whether the variation of pitch and corresponding SFOV affects image quality in the turbo FLASH mode third‐generation DSCT is still rarely reported.

The aim of this study was to investigate the effect of different pitches and the corresponding SFOVs on the image quality in the ultrafast, high‐pitch turbo FLASH mode third‐generation dual‐source CT using phantom.

## 2 MATERIALS AND METHODS

### 2.1 Experimental subject

**FIGURE 1:**
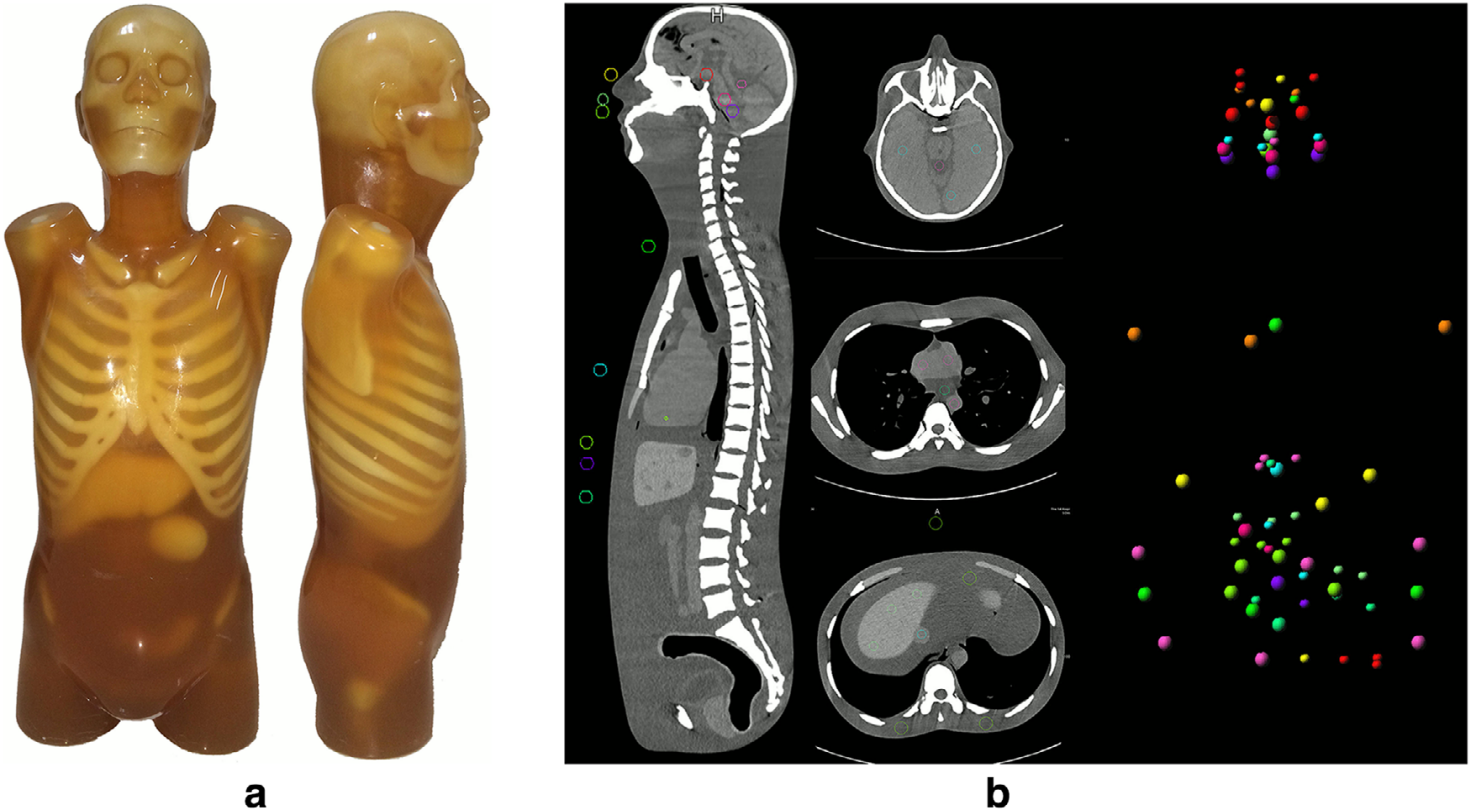
(a) Kyoto Kagaku CTU‐41 phantom. (b) Spherical regions of interest (ROIs) were positioned in the head, chest, and abdomen

This phantom study was exempted from the institutional review board approval. A head‐neck‐torso anthropomorphic CT Phantom [(CTU‐41; Kyoto Kagaku Co., Ltd., Kyoto, Japan); height, 100 cm; weight, 45 kg; phantom materials, materials with the X‐ray absorption rate equivalent to that of the human tissues] was selected to simulate the tissues and organs from head to the pelvis of an adult man. The phantom was verified at delivery to ensure that the CT numbers of the tissues and organs were similar to those in the human body using a conventional CT 120 kV scanning. Therefore, it was considered appropriate to use in an actual clinical setting (Figure 1).

### 2.2 Instrument and procedure

All CT scans were performed on a 192‐section third‐generation DSCT (Somatom Force; Siemens Healthcare Sector, Erlangen, Germany) using an ultrafast, high‐pitch turbo FLASH protocol. The scanning parameters were: collimation of 2 × 192 × 0.6 mm with the z‐flying focal spot technique and gantry rotation time of 0.25 s. Image acquisition was performed in the craniocaudal direction. The scan length included the entire head, chest, abdomen, and pelvis, from the top of the head to the lower edge of the pubic symphysis.

The different series of images were evaluated both objectively and subjectively. From the objective aspect, the CT number, image noise, signal‐to‐noise ratio (SNR), contrast‐to‐noise ratio (CNR), and artifacts index (AI) were compared between images with a minimum pitch of 1.55 and SFOV of Ø 50 cm and a maximum pitch of 3.2 and SFOV of Ø 35.4 cm. To verify the influence of different tube voltages on the objective image quality, the lowest tube voltage of 70 kV, the middle voltage of 120 kV (mostly used), and the highest tin‐filtered voltage of 150 kV (Sn 150 kV) were used. With the use of pitch 3.2 in the turbo FLASH mode, the maximum effective tube current‐time products (Eff.mAs_max_) were 202 mAs for 70 kV (70 kV/202 mAs), 36 mAs for 120 kV (120 kV/36 mAs), and 74 mAs for Sn 150 kV (Sn 150 kV/74 mAs) to keep the volume CT dose index (CTDI_vol_) constant of 1.8 mGy. Furthermore, 120 kV/68 mAs and 120 kV/136 mAs combinations were used to evaluate the objective image quality with different tube potential (CTDI_vol_) of 3.6 and 7.2 mGy. Each of the combinations was scanned three times. From the subjective aspect, 18 series of CT images with the different pitches and corresponding SFOVs (pitches: 1.55 to 3.2 with an increment of 0.1; SFOVs: 50 cm to 35.4 cm) were captured using 120 kV/136 mAs at 7.2 mGy CTDI_vol_ and independently evaluated by three radiologists.

All images were reconstructed to 1.5 mm section thickness with a 1.5 mm increment. To eliminate the influence of the display field of view (DFOV) on the image spatial resolution and ensure the accuracy of region of interest (ROI) copy‐and‐paste function, the reconstruction parameters were as follows: a matrix size of 512 × 512 pixel, a DFOV of 354 mm, use of a medium‐smooth soft‐tissue kernel (Br40), an iterative reconstruction algorithm ADMIRE, and Strength 3 (Advanced Modeled Iterative Reconstruction, Siemens Healthcare, Forchheim, Germany).

### 2.3 Image analysis

All images were analyzed using a research‐prototype radiomics software (Radiomics V1.2.3, Siemens Healthcare). The head, chest, and abdomen images of the phantom were evaluated. For each body part, three typical slices including the upper, middle, and lower fields were selected from the same tissue. First, four spherical ROIs (Ø 10 mm) were measured at each slice, and three of them were positioned on the soft tissues like brain parenchyma, mediastinum, and liver parenchyma, and the other ROI was positioned on the low‐density ventricle or blood vessel (Figure 1b). The mean CT number of the three soft‐tissue ROIs (1.47 ml) was defined as HU_soft tissue_, and its standard deviation (SD) was defined as SD_soft tissue_, and the SD of the low‐density ROI (0.47 ml) was defined as the CT background image noise (SD_background_). The SNR and CNR were calculated as

(1) $$\mathrm{SNR}=HU_{\text{soft}\;\text{tissue}}/SD_{\text{soft}\;\text{tissue}}$$

(2) $$\mathrm{CNR}\;=\;\left(HU_{\mathrm{soft}\;\mathrm{tissue}}\;-\;HU_{\mathrm{low}-\mathrm{density}}\right)/\;SD_{\mathrm{background}}$$

Second, the other three slices from each part that had significant artifacts were selected to calculate the AI.^23^ Four spherical ROIs (Ø 15 mm) were used, and three of them were positioned on the peripheral soft tissues and the other ROI was positioned on the air away in the scan field. The mean SD of the three peripheral soft‐tissue ROIs (4.81 ml) was defined as SD_peripheral soft tissues_, and the AI was calculated as

(3) $$\mathrm{AI}\;=\sqrt{{\mathrm{SD}}_{\mathrm{peripheral}\;\mathrm{soft}\;\mathrm{tissue}}^{2}-{\mathrm{SD}}_{\mathrm{Air}}^{2}}$$

The noise power spectrum can be used to evaluate the image texture,^23^ but it cannot be used to evaluate image texture when the anthropomorphic CT Phantom is used. The radiomics features included the shape features, first‐order features, and texture features. First‐order features describe the distribution of values of individual voxels without concern for the spatial relationships, whereas the texture features describe statistical interrelationships between voxels with similar or dissimilar contrast values.^24^ To reveal the differences of the image features between the pitch of 1.55 and SFOV of Ø 50 cm and the pitch of 3.2 and SFOV of Ø 35.4 cm, 837 image features including 162 first‐order features and 675 texture features from each soft‐tissue ROI (1.47 ml) were analyzed using the radiomics software (Radiomics V1.2.3, Siemens Healthcare) in each slice. Finally, we obtained a total of 45 ROIs from each body part (5 potential combinations × 3 times repeated scan × 3 slices) independently.

For the subjective assessment, the data sets of 18 series of the CT images of head, chest, and abdomen were obtained using different pitches and corresponding SFOVs and assessed independently by three radiologists with 15, 15, and 5 years of experience on a workstation (Syngo.via VB20A, Siemens Healthcare Sector), respectively (blinded for review). The data sets were randomized, and the three readers were blinded to the acquisition parameters. Because of the diverse structures in the head, chest, and abdomen, free adjustment of window width and level was approved. There were no lesions in this anthropomorphic phantom, and, therefore, the subjective analysis was performed by using the 3‐point and 5‐point Likert scales. Two sections were included: artifacts (3‐point scale—1: severe artifacts affecting visualization of major structures; 2: moderate artifacts not affecting visualization of major structures; 3: minimal artifacts); anatomical structures (5‐point scale—1: nondiagnostic examination; 2: major structures were moderately blurred and the diagnosis was only for a limited clinical situation such as calcified or large lesions; 3: major structures were slightly blurred and the diagnosis was still possible; 4: major structures were clear and the diagnosis was probably confident; 5: excellent).

### 2.4 Statistical analysis

Statistical analyses were performed using commercially available software programs (PRISM release 8.4, GraphPad Software, LLC; SPSS, release 26, SPSS Inc.; Python, Anaconda 2020.11, Anaconda Inc.). The measurement data were expressed as mean and SD. The data were tested for normal distribution using the Kolmogorov‐Smirnov test. The HU_soft tissue_, SD_background_, SNR, CNR, and AI were analyzed using a two‐tailed Student paired *t* test between the pitch of 1.55 and SFOV of Ø 50 cm and the pitch of 3.2 and SFOV of Ø 35.4 cm. Each soft‐tissue ROI has 837 image features including 162 first‐order features and 675 texture features, and 45 ROIs × 837 image features from each body part were tested using a two‐tailed Student paired *t* test between the pitch of 1.55 and SFOV of Ø 50 cm and the pitch of 3.2 and SFOV of Ø 35.4 cm images using Python, the Anaconda software. The *P* value of <0.05 was considered statistically significant. Interobserver agreement on the subjective image quality was calculated using multirater Fleiss kappa statistics (excellent agreement, *k *= 1.0–0.81; good agreement, *k *= 0.80–0.61; moderate agreement, *k *= 0.60–0.41; fair agreement, *k *= 0.40–0.21; poor agreement, *k *= 0.20–0.0).^25^

## 3 RESULTS

### 3.1 Relationship between pitch, SFOV, and tube potential

**FIGURE 2:**
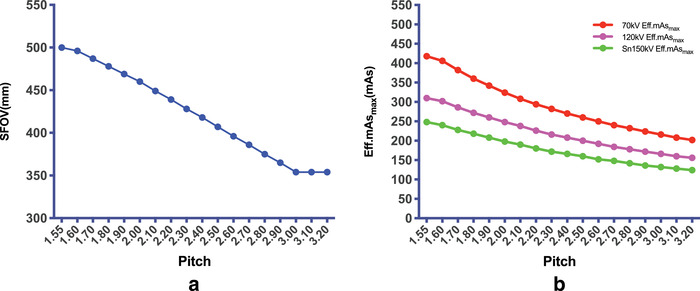
(a) The maximum scan field of view (SFOV) in the turbo FLASH model was 50 cm when the pitch was 1.55 and the SFOV decreased to 35.4 cm when the pitch was increased to 3.2. (b) The Eff.mAs_max_ of 70 kV, 120 kV, and Sn 150 kV were decreased when the pitch was increased from 1.55 to 3.2

The maximum SFOV in the turbo FLASH model was 50 cm when the pitch was 1.55 and the SFOV decreased to 35.4 cm when the pitch was increased to 3.2 (Figure 2a). The Eff.mAs_max_ of 70 kV, 120 kV, and Sn 150 kV were decreased when the pitch was increased from 1.55 to 3.2, and the Eff.mAs_max_ was decreased with the increase of tube voltage (Figure 2b).

### 3.2 Objective image quality

There was no significant difference in the CT number of the chest, abdomen, and part of the head between the pitch of 1.55 and SFOV of Ø 50 cm and the pitch of 3.2 and SFOV of Ø 35.4 cm images (*p *> 0.05), except for the 70 kV with 202 mAs, 120 kV with 136 mAs, and Sn 150 kV with 74 mAs for the head (*p *< 0.05). Image noises of the CT images with the pitch of 1.55 were lower than those with the pitch of 3.2, except for the 70 kV with 202 mAs combination in the head. The image noises between the two different pitches were statistically significant for the most body part (*p *< 0.05), except for the 70 kV with 202 mAs and 120 kV with 136 mAs combinations in the head, Sn 150 kV with 74 mAs combination in the chest, 120 kV with 34 and 136 mAs, and Sn 150 kV with 74 mAs combinations in the abdomen (*p *> 0.05). The SNR of the CT images with the pitch of 1.55 was slightly higher than that with the pitch of 3.2 images, and the SNR between the two different pitches were statistically significant for the 70 kV with 202 mAs, 120 kV with 68 mAs, and 136 mAs combinations in the head, 120 kV with 68 and 136 mAs in the chest, 70 kV with 202 and 120 kV with 68 mAs combinations in the abdomen (*p *< 0.05). The CNR of the CT images with the pitch of 1.55 images was slightly higher than that with the pitch of 3.2 images, and the CNR between the two different pitches were statistically significant for the 120 kV with 34 and 68 mAs in the head, 120 kV with 68 and 136 mAs in the chest, and 70 kV with 202 mAs and 120 kV with 68 mAs in the abdomen (*p *< 0.05). The AI of the CT images with the pitch of 1.55 was lower than that with the pitch of 3.2 images, and the AI between the two different pitches were statistically significant in the abdomen and for 120 kV with 68 mAs and 136 mAs combinations in the head and chest (*p *< 0.05) (Table 1).

**TABLE 1:** The objective image quality between the pitch of 1.55 and scan field of view (SFOV) of Ø 50 cm and the pitch of 3.2 and SFOV of Ø 35.4 cm; quantitative data were expressed as mean ± SD

Scan protocol					Head					Chest					Abdomen				
Pitch	SFOV (cm)	DFOV (cm)	kV	mAs	CT value (HU)	SD_background_	SNR	CNR	AI	CT value (HU)	SD_background_	SNR	CNR	AI	CT value (HU)	SD_background_	SNR	CNR	AI
			**70**	**202**	32.21 ± 1.62^*^	12.32 ± 2.12	2.93 ± 0.55^*^	5.43 ± 0.78	20.27 ± 6.61	25.34 ± 4.16	13.51 ± 3.33*	2.40 ± 0.3	5.58 ± 1.35	21.00 ± 1.97	84.53 ± 2.76	20.85 ± 2.62^*^	4.12 ± 0.36^*^	2.87 ± 0.39^*^	15.95 ± 1.62^*^
			**120**	**34**	43.13 ± 1.55	10.74 ± 1.15^*^	4.58 ± 0.6	3.57 ± 0.37^*^	14.99 ± 3.92	38.82 ± 3.68	14.12 ± 5.45^*^	3.99 ± 0.41	3.54 ± 1.18	14.18 ± 2.1	75.17 ± 2.57	18.33 ± 1.92	4.40 ± 0.39	1.98 ± 0.23	12.68 ± 1.21^*^
**3.2**	**35.4**	**35.4**		**68**	43.00 ± 1.39	10.08 ± 1.98^*^	4.91 ± 1.03^*^	3.86 ± 0.85^*^	14.21 ± 3.96^*^	38.60 ± 2.42	13.32 ± 6.25^*^	4.33 ± 0.59^*^	3.81 ± 1.33^*^	13.46 ± 2.3^*^	75.19 ± 2.69	16.88 ± 3.06^*^	4.84 ± 0.98^*^	2.22 ± 0.49^*^	11.62 ± 1.94^*^
				**136**	42.22 ± 1.3^*^	5.79 ± 0.66	8.43 ± 1.28^*^	6.59 ± 0.79	12.50 ± 3.17^*^	38.04 ± 1.69	10.11 ± 6.23^*^	6.35 ± 1.05^*^	5.65 ± 1.94^*^	12.03 ± 2.53^*^	74.79 ± 2.27	9.92 ± 1.12	8.65 ± 0.79	3.77 ± 0.73	7.80 ± 1.86^*^
			**Sn150**	**74**	46.14 ± 1^*^	10.87 ± 1.41^*^	4.97 ± 0.61	2.11 ± 0.39	10.07 ± 3.27	46.53 ± 1.33	13.35 ± 4.21	4.63 ± 0.42	2.00 ± 0.63	11.76 ± 2.14^*^	69.39 ± 2.7	17.48 ± 1.76	4.38 ± 0.32	1.34 ± 0.14	10.97 ± 0.5^*^
			**70**	**202**	33.53 ± 1.81^*^	12.45 ± 2.54	3.16 ± 0.59^*^	5.40 ± 1	19.53 ± 3.86	22.87 ± 0.72	10.94 ± 1.38^*^	2.46 ± 0.31	6.45 ± 0.66	20.20 ± 1.6	82.52 ± 1.43	17.69 ± 2.1^*^	4.69 ± 0.34^*^	3.35 ± 0.53^*^	13.40 ± 1.02^*^
			**120**	**34**	43.74 ± 0.62	10.16 ± 1.19^*^	4.67 ± 0.8	3.78 ± 0.47^*^	13.95 ± 2.28	37.55 ± 1.2	10.60 ± 1.63^*^	4.19 ± 0.37	4.13 ± 0.38	12.61 ± 1.02	75.26 ± 0.98	18.02 ± 1.73	4.49 ± 0.36	2.01 ± 0.21	11.59 ± 0.63^*^
**1.55**	**50**	**35.4**		**68**	43.70 ± 0.69	7.77 ± 0.87^*^	6.45 ± 0.93^*^	4.90 ± 0.48^*^	12.22 ± 2.63^*^	38.03 ± 0.81	7.98 ± 1.34^*^	5.97 ± 0.65^*^	5.66 ± 0.67^*^	10.28 ± 0.91^*^	75.53 ± 0.99	13.27 ± 1.51^*^	6.22 ± 0.55^*^	2.78 ± 0.39^*^	8.68 ± 1.36^*^
				**136**	43.93 ± 0.66^*^	5.55 ± 0.65	9.06 ± 1.08^*^	6.89 ± 0.58	11.42 ± 2.66^*^	38.12 ± 0.9	5.95 ± 1.15^*^	7.99 ± 0.47^*^	7.68 ± 1.05^*^	9.71 ± 0.8^*^	75.10 ± 1.12	9.55 ± 1.15	8.80 ± 0.72	3.86 ± 0.61	6.26 ± 1.56^*^
			**Sn150**	**74**	47.04 ± 0.47^*^	10.09 ± 0.99^*^	5.08 ± 0.57	2.23 ± 0.33	9.6 ± 1.95	46.39 ± 1.02	11.01 ± 1.61	4.96 ± 0.4	2.66 ± 0.14	10.03 ± 2.04^*^	69.81 ± 1.39	17.47 ± 2.68	4.47 ± 0.51	1.38 ± 0.12	10.12 ± 0.25^*^

Abbreviations: AI, artifacts index; CNR, contrast‐to‐noise ratio; HU, Hounsfield unit; SNR, signal‐to‐noise ratio.

Statistical significance (*p *< 0.05).

**FIGURE 3:**
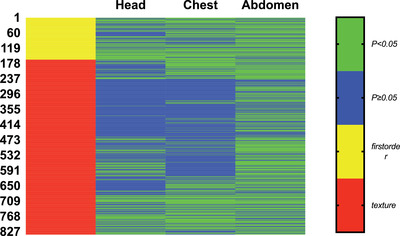
One hundred sixty‐two first‐order features (yellow area) and 675 texture features (red area) were compared between the pitch of 1.55 and scan field of view (SFOV) of Ø 50 cm and the pitch of 3.2 and SFOV of Ø 35.4 cm from each body part. There were significant differences in first‐order features and texture features of head (59.3%, 28.3%), chest (66%, 35.7%), and abdomen (71.6%, 64.7%) (*p *< 0.05)

A total of 837 image features from the 45 ROIs of each body part were analyzed. For the image features for the head soft tissues, 59.3% first‐order features and 28.3% texture features between the pitch of 1.55 and SFOV of Ø 50 cm and the pitch of 3.2 and SFOV of Ø 35.4 cm images were statistically different (*p *< 0.05). For the chest soft tissues, 66% first‐order features and 35.7% texture features between the two different pitches were statistically different (*p *< 0.05). For the abdominal soft tissues, 71.6% first‐order features and 64.7% texture features between the two different pitches were statistically different (*p *< 0.05) (Figure 3).

### 3.3 Subjective image quality

The subjective evaluation of the 18 series of images from the head, chest, and abdomen using different pitches from 1.55 to 3.2 is presented in Table 2. For the images from the head, the artifacts were minimal when the pitch was less than 2.0 and the artifacts were increased with the increase of pitch. The visualization of major structures was affected when the pitch was greater than 3.0 (*k *= 0.8). The anatomical structures were excellent when the pitch was less than 2.0, then decreased with the increase of the pitch, and major structures were moderately blurred when the pitch was greater than 3.0 (*k *= 0.69). For the images from the chest, the artifacts were minimal when the pitch was less than 2.3, then increased with the increase of pitch, and the visualization of major structures was affected when the pitch was greater than 3.0 (*k *= 0.7). The anatomical structures were excellent when the pitch was less than 2.0, then decreased with the increase of pitch, and the major structures were moderately blurred when the pitch was greater than 3.0 (*k *= 0.73). For the images from the abdomen, the artifacts were minimal when the pitch was less than 2.9, then increased with the increase of pitch, and the visualization of major structures was affected when the pitch was greater than 3.0 (*k *= 0.69). The anatomical structures were excellent when the pitch was less than 2.3, then decreased with the increase of pitch, major structures were slightly blurred, and the diagnosis was still possible when the pitch was greater than 3.0 (*k *= 0.76).

**TABLE 2:** The subjective image quality of the 18 series of images from the head, chest, and abdomen using different pitches from 1.55 to 3.2

	Head		Chest		Abdomen	
	Artifacts	Anatomical structure	Artifacts	Anatomical structure	Artifacts	Anatomical structure
Pitch	(Observer1,2,3)	(Observer1,2,3)	(Observer1,2,3)	(Observer1,2,3)	(Observer1,2,3)	(Observer1,2,3)
1.55	(3,3,3)	(5,5,5)	(3,3,3)	(5,5,5)	(3,3,3)	(5,5,5)
1.6	(3,3,3)	(5,5,5)	(3,3,3)	(5,5,5)	(3,3,3)	(5,5,5)
1.7	(3,3,3)	(5,5,5)	(3,3,3)	(5,5,5)	(3,3,3)	(5,5,5)
1.8	(3,3,3)	(5,5,5)	(3,3,3)	(5,4,5)	(3,3,3)	(5,5,5)
1.9	(3,2,3)	(5,5,5)	(3,3,3)	(5,4,5)	(3,3,3)	(5,5,5)
2.0	(2,2,3)	(4,3,5)	(3,2,3)	(4,4,5)	(3,3,3)	(5,5,5)
2.1	(2,2,2)	(4,3,4)	(3,2,3)	(4,4,4)	(3,3,3)	(5,5,5)
2.2	(2,2,2)	(4,3,4)	(3,2,3)	(4,4,4)	(3,3,3)	(5,5,5)
2.3	(2,2,2)	(3,3,3)	(2,2,2)	(4,4,4)	(3,3,3)	(4,5,4)
2.4	(2,2,2)	(3,3,3)	(2,2,2)	(4,4,4)	(3,3,3)	(4,4,4)
2.5	(2,2,2)	(3,3,3)	(2,2,2)	(4,4,4)	(3,3,3)	(4,4,4)
2.6	(2,2,2)	(3,3,3)	(2,2,2)	(4,4,4)	(3,3,3)	(4,4,4)
2.7	(2,2,2)	(3,3,3)	(2,2,2)	(4,3,4)	(3,3,3)	(4,4,4)
2.8	(2,2,2)	(3,3,3)	(2,2,2)	(3,3,3)	(3,3,3)	(4,3,4)
2.9	(2,2,2)	(3,3,3)	(2,1,2)	(3,3,3)	(2,2,2)	(4,3,4)
3.0	(2,2,1)	(3,3,2)	(2,1,1)	(3,2,2)	(2,2,1)	(4,3,3)
3.1	(1,1,1)	(2,2,2)	(1,1,1)	(2,2,2)	(1,2,1)	(3,3,3)
3.2	(1,1,1)	(2,2,2)	(1,1,1)	(2,2,2)	(1,2,1)	(3,3,3)
***k***	**0.8**	**0.69**	**0.7**	**0.73**	**0.69**	**0.76**

*Note*: Artifacts (3‐point scale: 1, severe artifacts affecting visualization of major structures; 2, moderate artifacts not affecting visualization of major structures; 3, minimal artifacts); anatomical structures (5‐point scale: 1, nondiagnostic examination; 2, major structures were moderately blurred and the diagnosis was only for a limited clinical situation such as calcified or large lesions; 3, major structures were slightly blurred and the diagnosis was still possible; 4, major structures were clear and the diagnosis was probably confident; 5, excellent).

## 4 DISCUSSION AND CONCLUSION

The key feature of the high‐pitch mode DSCT is its improved temporal resolution.^26^ The third‐generation DSCT turbo FLASH mode temporal resolution can be up to 66 ms with the highest pitch of 3.2 and SFOV of Ø 35.4 cm, and it is suitable for conditions requiring high temporal resolution and no large SFOV, like cardiovascular CT imaging,^8^, ^27^, ^28^, ^29^ and nonobese patient or pediatrics imaging.^1^, ^30^, ^31^ Because the scanning speed of the third‐generation turbo FLASH mode is more ultrafast than the second‐generation FLASH mode, using the turbo FLASH mode with a lower pitch can still achieve a similar time resolution when using the FLASH mode with a higher pitch. In addition, the SFOV of the turbo FLASH mode can be increased from Ø 35.4 to Ø 50 cm with the decrease of the pitch from 3.2 to 1.55. This improvement greatly expands the scope of the clinical evaluation, especially for obese patients. Besides, some applications need both temporal resolution and SFOV. Agostini et al. investigated the CT images of 75 patients confirmed with the COVID‐19 and found that using a turbo FLASH model with a pitch of 2.5 and SFOV of Ø 40.7 cm significantly reduced the radiation dose and motion artifacts.^18^ Therefore, the turbo FLASH model with a higher pitch could be used to improve the temporal resolution and reduce the acquisition time, and the lower pitch could be used to expand the SFOV to be adapted to obese patients. In the conventional CT, it has been approved that using the high‐pitch scan can affect the image quality.^21^ With the second‐generation DSCT, Flohr et al.^22^ found that the artifacts were more prevalent for the high‐pitch 3.2 scan mode and the structures varied markedly along the z‐axis, particularly for the head scans. However, whether the variation of pitch and corresponding SFOV affect the image quality in the third‐generation turbo FLASH mode is still rarely reported.

In our study, we compared the objective image qualities such as the CT number, image noise, SNR, CNR, and AI between the CT images with the pitch of 1.55 and SFOV of Ø 50 cm and those with the pitch of 3.2 and SFOV of Ø 35.4 cm and compared the image features between them using the radiomics software in an anthropomorphic phantom creatively. To our knowledge, no study investigated the image quality by the ultrafast, high‐pitch turbo FLASH mode with varying pitch and corresponding SFOV using the third‐generation DSCT.

We found that the differences in the CT numbers were not statically significant between the CT images with the pitch of 1.55 and SFOV of Ø 50 cm and those with the pitch of 3.2 and SFOV of Ø 35.4 cm from the most body parts and potential combinations (*p *> 0.05), except a slight difference for some potential combinations in the head (*p *< 0.05). These findings demonstrated that the variation of pitch and corresponding SFOV did not affect the CT number, which can be used for the quantitative analysis in the turbo FLASH mode. The image noises of the CT images with the pitch of 1.55 were lower than those with the pitch of 3.2. The SNR and CNR of the CT images with a pitch of 1.55 were slightly higher than those with a pitch of 3.2, which showed that the image quality by a lower pitch was still a little better than that by a higher pitch in the turbo FLASH mode. The AI of the CT images with the pitch of 1.55 images was lower than that with the pitch of 3.2, and the AI between the two different pitches was statistically different for the abdomen (all *P *< 0.05), 120 kV with 68 mAs and 136 mAs combinations in the head and chest, and Sn 150 kV with 74 mAs combination in the chest (*p *< 0.05), which demonstrated that the turbo FLASH mode still produced more artifacts with the increasing pitch, which was similar to the second‐generation DSCT.^22^

Comparing the image features between the pitch of 1.55 and SFOV of Ø 50 cm and the pitch of 3.2 and SFOV of Ø 35.4 cm images, we found that the first‐order features and texture features were significantly different in the images from the head, chest, and abdomen. These results showed that variation in the pitch and corresponding SFOV changed the image features in the third‐generation turbo FLASH mode, especially in the images from the abdomen. Because the DSCT FLASH mode uses two sets of projection data to combine to produce a complete (180°) sinogram, the proportion of the two data sets was constantly adjusted with the variation of pitch and SFOV,^26^, ^32^ and it may affect the image features. In addition, the density and structures in the head, chest, and abdomen were different; therefore, the different image noises were generated with the same scanning parameters in the different parts, and this variation may explain why the comparison results were different in the head, chest, and abdomen. So, the image features differed between the pitch of 1.55 and SFOV of Ø 50 cm and the pitch of 3.2 and SFOV of Ø 35.4 cm images in the turbo FLASH mode.

**FIGURE 4:**
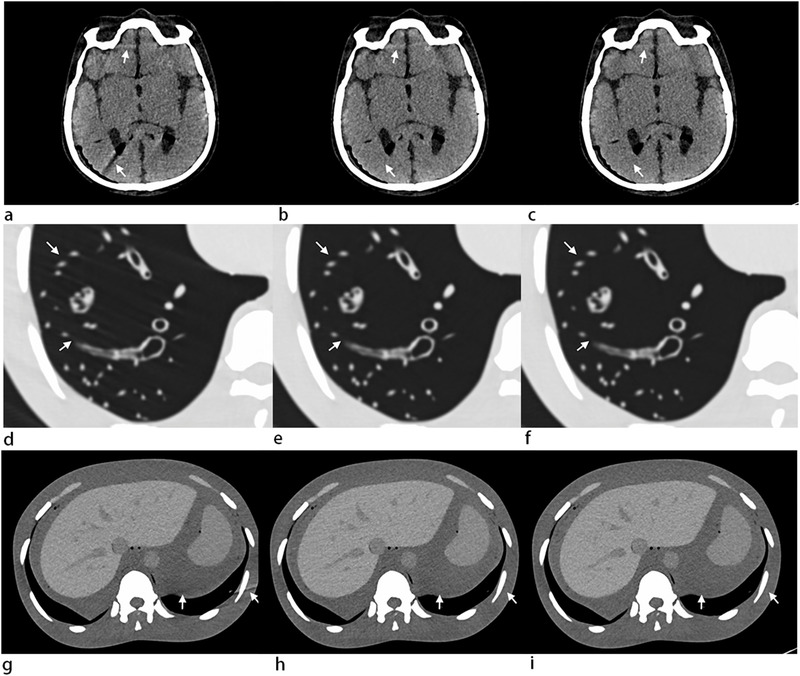
Nine of 18 series images obtained using different pitch from 1.55 to 3.2 for the head, chest, and abdomen; all images were obtained with tube voltage being set at 120 kV with 136 mAs. (a–c) Head images with pitch of 3.2, 1.9, and 1.55; (d–f) chest images with pitch of 3.2, 2.1, and 1.55; (g–i) abdomen images with pitch of 3.2, 2.8, and 1.55. The image quality of the pitch of 3.2 with scan field of view (SFOV) (Ø 35.4 cm) images decreased significantly (marked by the white arrow)

From the perspective of the subjective evaluation, 18 series of images of the head, chest, and abdomen were obtained using the different pitches from 1.55 and SFOV of Ø 50 cm to 3.2 and SFOV of Ø 35.4 cm. The image quality was excellent when the pitch was less than 2.0 and gradually decreased with the increase in pitch. In addition, the image quality decreased significantly when the pitch was higher than 3.0, and the increase of the pitch affected the image quality obviously in the head and chest than in the abdomen (all *k* ≥ 0.69) (Figure 4). This observation suggested that the higher pitch scan produced more artifacts, typically because there were rapid changes along the z‐axis of highly attenuating materials, which was consistent with the results of Flohr et al.^22^

In the DSCT FLASH mode, the sampling gaps of detector A caused by the high pitch are filled with the data acquired by detector B, and the pitch can be increased from 1.55 up to 3.2.^26^, ^32^, ^33^ In the third‐generation DSCT gantry design, detector A covers the whole SFOV of Ø 50 cm and detector B is restricted to the SFOV of Ø 35.5 cm because of the limited technology. The lower pitch can extend the SFOV in the third‐generation turbo FLASH mode, which differs from the second‐generation DSCT FLASH mode of which the SFOV is limited to the Ø 33 cm all the time. Meanwhile, the extended SFOV uses data A to extrapolate the data B at a certain projection angle θ, which is acquired either a quarter rotation earlier or later.^26^ When using the highest pitch of 3.2 and SFOV of Ø 35.4 cm, no redundant data were acquired by detector A and detector B. With the decrease in the pitch, the temporal resolution worsens, but more redundant data were acquired increasing the angular data segment that corresponds to an image.^26^, ^33^ This principle may explain the decrease of the image quality of the turbo FLASH model with the increase of the pitch. Due to the rapid changes along the z‐axis of high‐attenuation materials in the head and chest, helical artifacts, beam hardening, data truncation, and cross‐scattered radiation artifacts which were corrected by the adequate algorithm,^34^ these artifacts worked together and had an impact on the image quality with an increase of the pitch in our study. Therefore, we should select an appropriate pitch and corresponding SFOV, which not only depends on the patient size but also needs to meet the clinical requirement and avoid severe artifacts.

In addition, the Eff.mAs_max_ for 70 kV, 120 kV, and Sn 150 kV were decreased with an increase of the pitch from 1.55 to 3.2, and the Eff.mAs_max_ was decreased with the increasing tube voltage (Figure 2b). Therefore, it is necessary to appropriately lower the pitch to ensure the stability of the tube potential and obtain the appropriate noise level when using the turbo FLASH model.

There are some limitations to our study. First, we did not evaluate the effect of motion artifact for the respiratory and cardiovascular pulsations using the different pitches with this phantom. Second, although the anthropomorphic phantom can be used to simulate the tissues, organs, and microenvironment of the human body, the differences still exist compared with a real organism, but there is no ethical risk in repeated scanning when a phantom is used. Third, this study did not discuss the effects of SFOV/pitch on the spatial resolution using this phantom, and therefore, further research will focus on the spatial resolution using another phantom. Fourth, to keep the CTDI_vol_ constant with different tube voltages, the radiation dose may not be appropriate when scanning different body parts, but the purpose of this study was to investigate the effect of different pitches and corresponding SFOVs on the image quality using the ultrafast, high‐pitch turbo FLASH mode, and all scanning based on the same standards illustrated the problem.

Our study concluded that increasing the pitch and lowering the corresponding SFOV will change the image features and cause more artifacts degrading the image quality in the ultrafast, high‐pitch turbo FLASH mode of the third‐generation DSCT. Specific to the clinical needs, decreasing the pitch not only expands the SFOV but also improves the image quality when the turbo FLASH model is used.

## CONFLICT OF INTEREST

The authors declare no conflict of interest.

## FUNDING INFORMATION

None.

## AUTHOR CONTRIBUTION

Guarantor of integrity of the entire study: Yang Zhou; study concepts: Yang Zhou, Zhiwei Zhang, and Lei Hu; study design: Yang Zhou and Zhiwei Zhang; definition of intellectual content: Yang Zhou; literature research: Yang Zhou; experimental studies: Yang Zhou, Lei Hu, and Rui Jin; data acquisition: Yang Zhou, Lei Hu, and Rui Jin; data analysis: Yang Zhou, Rui Jin, and Silin Du; statistical analysis: Yang Zhou and Lei Hu; manuscript preparation: Yang Zhou; manuscript editing: Yang Zhou, Silin Du,and Wangjia Li; and manuscript review: Fajin Lv and Zhiwei Zhang.
